# Pineal gland volume is associated with prevalent and incident isolated rapid eye movement sleep behavior disorder

**DOI:** 10.18632/aging.102661

**Published:** 2020-01-09

**Authors:** Jeongbin Park, Ji Won Han, Seung Wan Suh, Seonjeong Byun, Ji Hyun Han, Jong Bin Bae, Jae Hyoung Kim, Ki Woong Kim

**Affiliations:** 1 Department of Brain and Cognitive Science, Seoul National University College of Natural Sciences, Seoul, Korea; 2 Department of Neuropsychiatry, Seoul National University Bundang Hospital, Seongnam, Korea; 3 Department of Radiology, Seoul National University Bundang Hospital, Seongnam, Korea; 4 Department of Radiology, Seoul National University College of Medicine, Seoul, Korea; 5 Department of Psychiatry, Seoul National University College of Medicine, Seoul, Korea

**Keywords:** pineal gland, RBD, MRI, aging, prospective

## Abstract

We aimed to investigate the association of pineal gland volume with the risk of isolated rapid eye movement (REM) sleep behavior disorder (RBD). We enrolled 245 community-dwelling cognitively normal elderly individuals without major psychiatric or neurological disorders at the baseline evaluation, of whom 146 completed the 2-year follow-up evaluation. We assessed RBD symptoms using the REM Sleep Behavior Disorder Screening Questionnaire (RBDSQ) and defined probable RBD (pRBD) as an RBDSQ score of ≥ 5. We manually segmented the pineal gland on 3T T1-weighted brain magnetic resonance imaging and estimated its volume. The smaller the baseline pineal gland volume, the more severe the RBD symptoms at baseline. The individuals with isolated pRBD showed smaller pineal gland volumes than those without isolated pRBD. The larger the baseline pineal gland volume, the lower the risks of prevalent isolated pRBD at the baseline evaluation and incident isolated pRBD at the 2-year follow-up evaluation. Pineal gland volume showed good diagnostic accuracy for prevalent isolated pRBD and predictive accuracy for incident isolated pRBD in the receiver operator characteristic analysis. Our findings suggest that pineal gland volume may be associated with the severity of RBD symptoms and the risk of isolated RBD in cognitively normal elderly individuals.

## INTRODUCTION

Rapid eye movement (REM) sleep behavior disorder (RBD) is a parasomnia characterized by the loss of normal skeletal muscle atonia during REM sleep and dream-enacting behaviors [1]. Its’ prevalence was estimated to be 1-2% in the general elderly populations [2, 3]. RBD can occur in association with (secondary RBD) or without (isolated RBD) a neurodegenerative disorder [1], and isolated RBD accounts for up to 60% at the diagnosis [4]. However, more than 80% of isolated RBD eventually developed a neurodegenerative disorder such as Parkinson’s disease (PD) and Lewy body disease (DLB) in 12-14 years [5, 6], which implies that a majority of isolated RBD may be a prodromal phase of α-synucleinopathies [1, 4].

A series of clinical trials found that the symptoms of RBD were improved by N-acetyl-5-methoxytryptamine (melatonin). In RBD patients, dream-enacting behaviors were reduced and REM sleep muscle atonia were restored by the administration of melatonin [7–11] but relapsed by discontinuation of melatonin [8]. Melatonin is a multifunctioning indoleamine produced by the pineal gland [12]. The pineal gland regulates sleep and circadian rhythm through the synthesis and secretion of melatonin [12, 13]. In humans, roughly 80% of the pineal gland is composed of melatonin-producing pinealocytes [13], and the volume of pineal gland (VPG) is proportional to the levels of melatonin in plasma, urine or saliva [14–16]. Although the pineal gland is reported to fully develop after the first year of life and does not change in size or weight later in life [12, 17, 18], recent studies have found that VPG could be changed by lifestyle such as coffee consumption or pathological conditions that may change melatonin production [19–21]. Given the effects of melatonin on RBD symptoms and the association of melatonin with VPG [14–16], we may assume that RBD patients may have smaller VPG than the individuals without RBD, and VPG may predict the future risk of RBD in the individuals without RBD. In this study, we investigated the association of VPG with current RBD symptoms cross-sectionally and with the future risk of RBD symptoms prospectively in cognitively normal (CN) elderly individuals without neurological or psychiatric disorders.

## RESULTS

The demographic and clinical characteristics of the participants are summarized in Table 1. The participants with probable RBD (pRBD) showed smaller volume of pineal parenchyma (VPP) and VPG than those without pRBD (p < 0.001).

**Table 1:** Demographic and clinical characteristics of the participants.

	**Prevalent pRBD at baseline**				**Incident pRBD at 2-year follow-up**		
	**Absent (n = 221)**	**Present (n = 24)**	**p**		**Absent (n = 142)**	**Present (n = 4)**	**p**
Age (years, mean ± SD)	71.79 ± 6.17	72.50 ± 6.16	0.594^a^		70.70 ± 5.87	76.00 ± 7.96	0.080^a^
Women, n (%)	114 (51.58)	11 (45.83)	0.592^b^		72 (50.70)	1 (25.00)	0.311^b^
Education (years, mean ± SD)	11.43 ± 4.93	10.42 ± 5.03	0.341^a^		11.35 ± 5.11	13.75 ± 4.03	0.352^a^
Presence of cohabitants, n (%)	191 (86.43)	23 (95.83)	0.188^b^		124 (87.32)	3 (75.00)	0.470^b^
Alcohol drinking (standard units/week, mean ± SD)	3.03 ± 7.66	6.00 ± 11.17	0.215^a^		3.67 ± 8.86	3.63 ± 5.71	0.992^a^
Smoking (packs/day, mean ± SD)	0.03 ± 0.18	0.02 ± 0.08	0.797^a^		0.04 ± 0.21	0.00 ± 0.00	0.680^a^
History of head injury, n (%)	11 (4.98)	1 (4.17)	0.861^b^		8 (5.63)	0 (0.00)	0.625^b^
MMSE (points, mean ± SD)	27.40 ± 2.06	27.17 ± 2.81	0.616^a^		27.29 ± 2.11	27.00 ± 0.82	0.786^a^
GDS (points, mean ± SD)	7.71 ± 5.63	9.93 ± 7.69	0.199^a^		7.49 ± 5.68	12.00 ± 6.38	0.121^a^
CIRS (points, mean ± SD)	5.62 ±2.83	6.33 ± 2.46	0.237^a^		5.09 ± 2.64	6.50 ± 3.42	0.298^a^
STOPBANG (points, mean ± SD)	2.54 ± 0.94	2.96 ± 0.75	0.037^a^		2.50 ± 0.95	3.25 ± 0.96	0.121^a^
RBDSQ (points, mean ± SD)							
Total score	1.48 ± 1.26	5.79 ± 1.22	< 0.001^a^		1.37 ± 1.21	3.00 ± 1.16	0.009^a^
Item 6 score	0.12 ± 0.37	1.17 ± 1.05	< 0.001^a^		0.10 ± 0.32	0.75 ± 0.96	0.267^a^
Intracranial volume (cm^3^, mean ± SD)	1565.40 ± 160.86	1553.13 ± 170.68	0.725^a^		1576.95 ± 156.49	1530.15 ± 66.05	0.553^a^
VPP (mm^3^, mean ± SD)	87.55 ± 30.04	58.42 ± 16.49	< 0.001^c^		90.13 ± 30.15	53.00 ± 20.07	0.016^a^
VPG (mm^3^, mean ± SD)	95.18 ± 39.96	61.13 ± 18.30	< 0.001^c^		98.58 ± 42.12	56.25 ± 20.99	0.048^a^

Abbreviations: pRBD = probable REM sleep behavior disorder; SD = standard deviation; MMSE = Mini-Mental State Examination; GDS = Geriatric Depression Scale; CIRS = Cumulative Illness Rating Scale; RBDSQ = REM Sleep Behavior Disorder Screening Questionnaire; VPP = pineal parenchyma volume; VPG = pineal gland volume.

^a^Independent sample t-test.

^b^Chi-square test.

^c^Analysis of covariance adjusted for age, sex, years of education, intracranial volume, head injury, amount of smoking, and amount of alcohol drinking.

**Figure 1:**
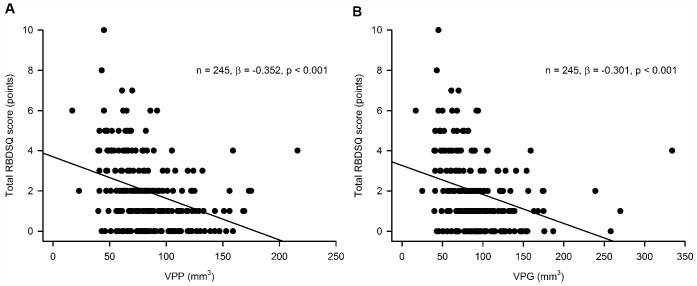
Association of (**A**) pineal parenchyma volume (VPP, mm^3^) and (**B**) pineal gland volume (VPG, mm^3^) with the REM Sleep Behavior Disorder Screening Questionnaire (RBDSQ) total score^a.^ ^a^Multiple linear regression model adjusted for age, sex, years of education, intracranial volume, head injury, amount of smoking, and amount of alcohol drinking.

Both VPP and VPG were inversely associated with the REM Sleep Behavior Disorder Screening Questionnaire (RBDSQ) total score (RBDSQ-T) (standardized β = -0.352, p < 0.001 for VPP; standardized β = -0.301, p < 0.001 for VPG) and the item 6 score of the RBDSQ (RBDSQ-6) (standardized β = -0.239, p < 0.001 for VPP; standardized β = -0.198, p = 0.002 for VPG), indicating that the individuals with smaller VPP or VPG may have more RBD symptoms (Figure 1). There was no evidence of multicollinearity in all regression models with the maximum variance inflation factor being 2.05.

Baseline VPP and VPG were inversely associated with the risk of prevalent pRBD at the baseline evaluation (odds ratio [OR] = 0.939, 95% CI = 0.912 - 0.966, p < 0.001 for VPP; OR = 0.947, 95% CI = 0.924 - 0.972, p < 0.001 for VPG), indicating that the individuals with larger VPP and VPG may have a lower risk of prevalent pRBD (Table 2). The baseline VPP and VPG were also inversely associated with the risk of incident pRBD at the 2-year follow-up evaluation (OR = 0.890, 95% CI = 0.798 - 0.993, p = 0.036 for VPP; OR = 0.912, 95% CI = 0.832 - 0.999, p = 0.047 for VPG), indicating that the individuals with larger VPP and VPG may have a lower risk of future pRBD (Table 2).

**Table 2:** Association of the baseline pineal parenchyma volume and pineal gland volume with the prevalent and incident probable REM sleep behavior disorder.

	**Total, n**	**pRBD, n**	**OR (95% CI)^a^**	**p^a^**
For prevalent pRBD				
VPP (mm^3^)	245	24	0.939 (0.912 - 0.966)	< 0.001
VPG (mm^3^)	245	24	0.947 (0.924 - 0.972)	< 0.001
For incident pRBD				
VPP (mm^3^)	146^b^	4	0.890 (0.798 - 0.993)	0.036
VPG (mm^3^)	146^b^	4	0.912 (0.832 - 0.999)	0.047

Abbreviations: pRBD = probable REM sleep behavior disorder; VPP = pineal parenchyma volume; VPG = pineal gland volume; OR = odds ratio; CI = confidence interval.

^a^Binary logistic regression analyses adjusting age, sex, years of education, intracranial volume, head injury, amount of smoking, and amount of alcohol drinking at the baseline evaluation as covariates.

^b^The number of participants who did not have probable RBD at the baseline evaluation and completed the 2-year follow-up evaluation.

**Figure 2:**
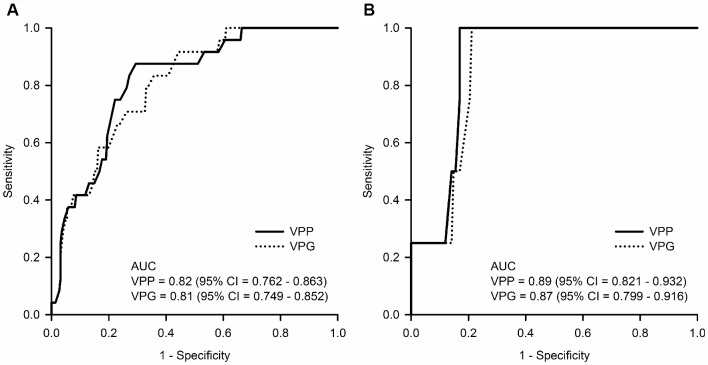
(**A**) Diagnostic accuracy for prevalent probable REM sleep behavior disorder (pRBD) at the baseline evaluation and (**B**) predictive accuracy for incident pRBD at the 2-year follow-up evaluation of the baseline pineal parenchyma volume (VPP, mm^3^) and pineal gland volume (VPG, mm^3^). Abbreviations: AUC = area under the receiver operator characteristic curve; CI = confidence intervals.

The diagnostic accuracies of the baseline VPP and VPG for prevalent pRBD at the baseline evaluation were good; area under the receiver operator characteristic curve (AUC) was 0.82 (95% CI = 0.762 - 0.863, p < 0.0001) for VPP and 0.81 (95% CI = 0.749 - 0.852, p < 0.0001) for VPG (Figure 2). The optimal cutoff values of the baseline VPP and VPG for classifying pRBD were 70 mm^3^ (sensitivity = 87.50%; specificity = 70.59%) and 77 mm^3^ (sensitivity = 83.33%; specificity = 64.25%), respectively. The predictive accuracies of baseline VPP and VPG for incident pRBD at the 2-year follow-up evaluation were also good; AUC was 0.89 (95% CI = 0.821 - 0.932, p < 0.0001) for VPP and 0.87 (95% CI = 0.799 - 0.916, p < 0.0001) for VPG (Figure 2). The optimal cutoff values of the baseline VPP and VPG for predicting incident pRBD at 2-year follow-up evaluation were 65 mm^3^ (sensitivity = 100.00%; specificity = 83.10%) and 69 mm^3^ (sensitivity = 100.00%; specificity = 78.87%), respectively.

## DISCUSSION

In animals, nighttime REM sleep disturbances were induced by lesioning the pineal gland, which reduced pineal melatonin secretion [22]. However, the association between the pineal gland and RBD has never been investigated in humans. The current study directly showed that smaller pineal gland was associated with the more RBD symptoms and the higher risk of incident pRBD in CN elderly individuals.

Considering that the pineal gland volume was highly correlated with the endogenous melatonin level [14–16], our observation seems to be in line with previous studies on the effects of melatonin on RBD. Melatonin treatment decreased the frequency and severity of dream-enacting behaviors and the risk of falls in the elderly RBD patients [10], and the beneficial effects of melatonin (3-12 mg) lasted beyond a year in a series of RBD patients [9]. A couple of clinical trials on the polysomnography-diagnosed RBD patients found that the percentage of REM sleep without atonia and movement time in REM were reduced by administering melatonin before bedtime for 4-6 weeks [7, 8] but relapsed by discontinuing melatonin [8]. Another open-label trial reported that the percentage of tonic REM activity was reduced from 16% to 6% by administering 3-9 mg of melatonin at night, especially in the elderly patients with low endogenous melatonin secretion [23]. A placebo-controlled trial also reported that the percentage of REM sleep increased from 14.7% to 17.8% and the clinical global impression and daytime dysfunction were improved by administering 3 mg melatonin before bedtime in the individuals with disturbed and reduced REM sleep duration [11]. However, it remains unknown how melatonin improves the RBD symptoms. Multiple actions of melatonin such as decreasing muscle tonicity during REM sleep, enhancing GABAergic inhibition, stabilizing circadian clock variability and desynchronization, protecting cytoskeletal structure through its antagonism of calmodulin, enhancing sleep efficiency and shortening sleep latency may underlie the its beneficial effect on RBD [1, 10]. In a glycine/GABA-A receptor knockout transgenic mouse model of RBD, melatonin was efficacious in decreasing REM motor behaviors and restoring REM muscle atonia [24].

VPP and VPG showed good diagnostic accuracy for prevalent pRBD and predictive accuracy for incident pRBD in the current study, indicating that VPP or VPG may be potential biomarkers of isolated RBD in cognitively normal elderly individuals. However, the reduced VPP or VPG of the elderly individuals with isolated pRBD or those who are destined to have isolated pRBD in the future may be associated with prodromal α-synucleinopathies rather than RBD itself. Indeed, PD patients showed diminished endogenous melatonin production compared to controls [25]. In addition, melatonin directly blocked the α-synuclein fibril formation, destabilized preformed α-synuclein fibrils, and decreased α-synuclein-induced cytotoxicity [26]. Nevertheless, the changes of pineal gland volume in the patients with α-synucleinopathies have not been directly investigated yet.

This study has a couple of strengths. First, we excluded the volume of pineal cysts (VPC) when we estimated the pineal parenchyma volume because pineal cysts do not contain pinealocytes [21, 27], and exclude the subjects with extremely large cystic glands from the current study. Although both VPP and VPG were associated with the prevalent and incident pRBD in the current study, pineal parenchyma volume (i.e., non-cystic volume) better reflected the levels of endogenous melatonin secretion than total pineal gland volume in adult individuals [14, 15]. Although we did not directly investigate the association between VPC and RBD, this result may suggest that the presence of the pineal cysts itself may not affect RBD symptoms. Second, we excluded the subjects who were depressive and/or were taking antidepressants since depression may increase the risk of RBD and antidepressants, selective serotonin reuptake inhibitor (SSRI) and serotonin norepinephrine reuptake inhibitor (SNRI) in particular, may produce dream-enacting behavior and loss of normal REM sleep atonia [1, 28]. However, this study also has several limitations. First, although the RBDSQ is well validated in polysomnography-confirmed RBD patients [1, 29], it may be subject to recall bias and misclassification bias. However, it seems unlikely that systematic errors would be made in reporting behavioral features of RBD since the current study included CN individuals who were examined only through comprehensive clinical and neuropsychological assessments. In addition, we tried to reduce the risk of misclassification bias by confirming the association of VPP and VPG with the RBDSQ item 6 score as well as RBDSQ total score. The RBDSQ item 6 queries the core symptoms of RBD with a good specificity [30]. Second, we cannot completely rule out the possibility that non-RBD symptoms might have influence the RBDSQ scores. For example, some patients with restless leg syndrome (RLS) or severe obstructive sleep apnea (OSA) showed the behaviors mimicking RBD during sleep [31, 32]. Although we exclude the participants with OSA by excluding the participants who got 5 points or higher in STOPBANG, the participants with pRBD showed modestly higher STOPBANG score than those without pRBD at baseline. Third, we could not adjust for the volume of pineal calcification because we did not perform additional brain computed tomography or high-resolution T2-weighted MRI. Although the effects of pineal calcification on melatonin production or human REM sleep remain unclear, a couple of previous studies argue that pineal calcification may inhibit the capacity for pineal melatonin synthesis and be associated with polysomnographic sleep parameters in humans [19, 33]. Furthermore, calcifications can appear hypointense on T1-weighted images, which may lead underestimation of the VPP. Fourth, although we excluded individuals who took exogenous melatonin over the past 6 weeks, we did not directly quantify the endogenous nocturnal melatonin levels in the blood. Fifth, the sample size was small. In the statistical power analysis with α error probability of 0.05 (G^*^Power version 3.1; http://www.gpower.hhu.de), the statistical power (1–beta error) for the prevalent and incident pRBD was 0.52 and 0.54, respectively. However, the association of pineal gland volume and the prevalent and incident pRBD was statistically significant despite the low statistical power in the current study. Sixth, since the small number of converters at 2-year follow-up, the accuracies of cutoff values of the baseline VPP and VPG for predicting incident pRBD may need to be further validated in future studies with a larger sample size and longer follow-up. Finally, the follow-up duration was short.

In summary, the smaller pineal gland was associated with the more current RBD symptoms and the higher future risk of RBD in cognitively normal elderly individuals, and VPP or VPG may be a candidate biomarker of RBD.

## MATERIALS AND METHODS

### Study participants

The present sample of 245 CN elderly individuals comprised 157 and 88 subjects from the participants of the baseline evaluation of the Korean Longitudinal Study on Cognitive Aging and Dementia (KLOSCAD) [34] and the first follow-up evaluation of the Korean Longitudinal Study on Health and Aging (KLOSHA) [35], respectively, which were conducted from November 2010 to October 2012. Both the KLOSCAD and KLOSHA were population-based prospective elderly cohort studies. The KLOSCAD study randomly sampled 30 villages and towns from 13 specific districts across South Korea, and randomly selected 10% and 20% of the elderly adults from urban and rural areas, respectively, using resident rosters and data on residents aged ≥ 60 years. The KLOSHA study randomly selected community-dwelling Korean elderly adults aged ≥ 65 years from the resident roster of Seongnam, one of the largest satellite cities of Seoul. Among the 221 participants who did not have pRBD at the baseline evaluation, 146 completed the 2-year follow-up evaluation. In the current study, we excluded the following conditions: cognitive disorders such as dementia and mild cognitive impairment (MCI); major psychiatric and/or neurologic disorders that could affect cognitive function; any history of brain tumors, substance abuse or dependence, and use of clonazepam, antidepressants (SSRI, SNRI, and others), or exogenous melatonin over the past 6 weeks; any serious medical condition that could affect the structure and/or function of the pineal gland or abnormalities in pineal gland morphology such as neoplastic lesions or extremely large cystic gland (diameter greater than 15.0 mm) [36]; and conditions that could mimic the symptoms of RBD such as RLS and OSA. We diagnosed RLS using the Cambridge-Hopkins Restless Leg Syndrome questionnaire (CHRLSq) [37] and defined OSA as a STOPBANG questionnaire [38] score of ≥ 5 points.

The study protocol was approved by the institutional review board of the Seoul National University Bundang Hospital. All the participants were fully informed of the study protocol, and written informed consent was provided by them or their legal guardians.

### Assessment of cognitive function

In both the KLOSCAD and the KLOSHA, geriatric psychiatrists with expertise in dementia research administered face-to-face standardized diagnostic interviews, detail medical histories, laboratory tests, and physical and neurological examinations using the Korean version of the Consortium to Establish a Registry for Alzheimer’s Disease Assessment Packet Clinical Assessment Battery (CERAD-K) [39] and the Korean version of the Mini International Neuropsychiatric Interview [40]. In addition, research neuropsychologists administered the Digit Span Test [41], Frontal Assessment Battery [42], Geriatric Depression Scale (GDS) [43], Cumulative Illness Rating Scale (CIRS) [44], and CERAD-K Neuropsychological Assessment Battery (CERAD-K-N) [39, 45]. All participants performed -1.0 SD of the age-, gender-, and education-adjusted norms of elderly Koreans on the Mini-Mental State Examination (MMSE) [46]. Using a study-specific standard interview, trained research nurses collected data on age, sex, years of education, intracranial volume (ICV), history of head injury, amount of smoking (packs/day), and alcohol drinking (standard units/week) over the past twelve months period.

A panel of research neuropsychiatrists determined the participants’ final diagnoses in both the KLOSCAD [34] and the KLOSHA [35]. Two neuropsychiatrists (K.W.K and J.W.H) participated in both panels. In both the KLOSCAD and the KLOSHA, we diagnosed dementia and other Axis I mental disorders according to the Diagnostic and Statistical Manual of Mental Disorders, 4^th^ Edition, Text Revision (DSM-IV-TR) criteria [47] and MCI according to the criteria by the International Working Group on MCI [48]. We defined CN as functioning independently in the community and showing no evidence of cognitive impairment in objective neuropsychological tests.

### Assessment of RBD symptoms

We evaluated behavioral features of RBD using the RBDSQ [29]. The RBDSQ is a self-reported screening instrument for diagnosing RBD and is comprised of 10 items assessing the most prominent clinical features of RBD: items 1 to 4, the frequency and content of dreams and their relationship to nocturnal movements and behavior; item 5, self-injuries and injuries to the bed partner; item 6, four subsections specifically assessing nocturnal motor behavior, e.g. questions about nocturnal vocalization (6.1), sudden limb movements (6.2), complex movements (6.3) or bedside items that fall down (6.4); items 7 and 8, nocturnal awakenings; item 9, disturbed sleep in general; and item 10, the presence of any neurological disorder. Each item could be answered as ‘‘yes’’ or ‘‘no’’. The RBDSQ score ranges from 0-13 points, with higher scores indicating more features associated with RBD. We defined pRBD individuals as having a total score of 5 or higher on the RBDSQ [29]. The questionnaire was completed by the subjects with aid from their partners if needed.

### Segmentation of the pineal gland

We performed brain MRI using a Philips 3.0 Tesla Achieva scanners (Philips Medical Systems; Eindohovenm, the Netherlands) within 3 months of the clinical assessments. We obtained 3D structural T1-weighted spoiled gradient echo sequences with the following parameters: acquisition voxel size = 1.0 × 0.5 × 0.5 mm; 1.0 mm sagittal slice thickness with no inter-slice gap; repetition time = 4.61 ms; echo time = 8.15 ms; number of excitations = 1; flip angle = 8°; field of view = 240 × 240 mm; and acquisition matrix size = 175 × 256 × 256 mm in the x-, y-, and z-dimensions. We implemented bias field correction to remove the signal intensity inhomogeneity artifacts of MR images using Statistical Parametric Mapping software (version 8, SPM8; Wellcome Trust Centre for Neuroimaging, London; https://www.fil.ion.ucl.ac.uk/spm) in MATLAB R2014a (MathWorks Inc., Natick, MA, USA). We resliced the MR images into an isotropic voxel size of 1.0 × 1.0 × 1.0 mm^3^. We measured ICV using FreeSurfer software (version 5.3.0; http://surfer.nmr.mgh.harvard.edu) to adjust for inter-individual variabilities in brain volume.

For each participant, trained researchers blinded to the demographics and clinical characteristics constructed a 3D mask of each pineal gland by manually segmenting the pineal gland slice-by-slice on the resliced T1-weighted MR images using the ITK-SNAP (version 3.4.0; http://www.itksnap.org) volumetric imaging software. We segmented the pineal glands primarily on the sagittal planes and corroborated the results on the axial and coronal planes. We identified the pineal gland using the following structures as guides: the quadrigeminal cisterna, posterior portion of the third ventricle, superior colliculus, and habenula. Except for the portion connected to the habenula, defining of the boundaries of the pineal gland was straightforward as it is surrounded by cerebrospinal fluid [13]. We carefully differentiated the pineal gland from the adjacent vascular structures, especially the vein of Galen and the paired internal cerebral veins. We defined a pineal cyst as an area of homogenous intensity that was isointense to cerebrospinal fluid in T1 sequence images [27, 49] with a diameter of 2.0 mm or greater [27]. We measured the VPG and VPC and estimated the VPP by subtracting VPC from VPG.

To determine inter-rater reliability, we assessed the intraclass correlation coefficients (ICCs) for 30 subjects who were randomly selected from the 258 participants with a time gap of 2 months. The ICCs were 0.971 (95% confidence interval [CI] = 0.940 - 0.986) for the VPG and 0.950 (95% CI = 0.894 - 0.976) for the VPC. The VPG was strongly correlated with the VPP (r = 0.914, p < 0.001) and the VPC (r = 0.701, p < 0.001) in our participants.

### Statistical analysis

We compared continuous variables using independent samples t-tests and categorical variables using chi-square tests between groups. We examined the associations of VPP and VPG with RBDSQ-T using multiple linear regression model adjusted for age, sex, years of education, ICV, head injury, smoking, and alcohol drinking as covariates. To test the robustness of our observation, we also examined the associations of VPP and VPG with the RBDSQ-6 using multiple linear regression model adjusted for diagnosis, age, sex, years of education, ICV, head injury, smoking, and alcohol drinking as covariates. In each of the linear regression models, VPP, VPG, RBDSQ-T and RBDSQ-6 were entered as continuous variables. We assessed multicollinearity using collinearity statistical tests (tolerance and variance inflation factor). We compared VPP and VPG between the participants with pRBD and those without pRBD using analysis of covariance that adjusted for age, sex, years of education, ICV, head injury, smoking, and alcohol drinking as covariates. We examined the association of baseline VPP and VPG with the risk of prevalent pRBD at baseline and the risk of incident pRBD at 2-year follow-up evaluation using binary logistic regression analyses that adjusted for age, sex, years of education, ICV, head injury, smoking, and alcohol drinking at the baseline evaluation as covariates. We examined the diagnostic performances of the baseline VPP and VPG for prevalent pRBD at the baseline evaluation and the predictive accuracy for incident pRBD at the 2-year follow-up evaluation using the ROC analyses. We calculated the optimal cutoff values and AUC using Youden index maximum (sensitivity + specificity − 1) [50].

For all analyses, we considered a two-tailed p-value less than 0.05 as statistically significant, and we employed Bonferroni corrections to reduce type I error when multiple comparisons were conducted. We performed all statistical analyses using SPSS for Windows (version 20.0; IBM Corporation; Armonk, NY).
